# Early Stopping Criterion for Recursive Least Squares Training of Behavioural Models

**DOI:** 10.1007/s11277-022-09813-9

**Published:** 2022-07-21

**Authors:** Méabh Loughman, Sinéad Barton, Ronan Farrell, John Dooley

**Affiliations:** Department of Electronic Engineering, Maynooth University, Co. Kildare, Ireland

**Keywords:** Behavioural modeling, Power Amplifier, Recursive least squares (RLS), Volterra Model

## Abstract

The necessity of the rapid evolution of wireless communications, with continuously increasing demands for higher data rates and capacity Zheng (Big datadriven optimization for mobile networks toward 5g 30:44–51, 2016), is constantly augmenting the complexity of radio frequency (RF) transceiver architecture. A significant component in the configuration of such complex radio transceivers is the power amplifier(PA). Multiple distributed PAs are now common in proposed RF architectures. PAs exhibit non linear behaviour, causing signal distortion in transmission. Behavioural models offer a concise representation of a PAs characteristic performance which is extremely useful in simulating performance of multiple nonlinear power amplifiers. A considerable drawback with using the Recursive Least Squares (RLS) technique is that the instability of the coefficients during the training of the model. This manuscript provides a computationally efficient technique to detect the onset of instability during adaptive RLS training and subsequently to inform the decision to cease training of dynamic memory polynomial based behavioural models, to avoid the onset of instability. The proposed technique does not require modification of the RLS algorithm, merely an observation of the pre-exsisting autocorrelation function based update. This technique is experimentally validated using four different signal modulation schemes, LTE OFDM, 5G-NR, DVBS2X and WCDMA.

## Introduction

Ideally a PA is an active device which linearly transforms an input signal, *x*, into an output signal, *y* such that $y = G x$. *G* is the gain experienced. PAs, in reality, illicit non linear behaviour and the linearity versus efficiency is a historical trade-off.

RF transceiver architecture now propose multiple parallel transmission paths, to incorporate high data throughput. The computation involved in modelling these increasingly complex systems has accelerated substantially. A considerable amount of work has been done to model many digital signal processing induced consequences such as by [2], authors utilise the statistical properties of the autocorrelation function to combat autoregressive fading , and adaptive clipping and selective mapping applied to achieve a distortion-less reduction of the average power of transmitted signals [3] .

Another prevalent modeling task which contributes to signal distortion on each signal path, is the PA. In PA modelling, RLS has been used in the past to train polynomial models with memory terms, however instability can occur when training the model weights.

The main objective in behavioural modelling RF systems is to identify the most computationally efficient structure that can accurately characterise the behaviour of such a complex non linear system.

Regardless of architecture, all PA’s operate as voltage controlled current sources. There are two categories in which PA behaviours can be subdivided.

1. Short term memory effects are caused by the frequency response of networks located in the matching networks and device parasitics.
2. Long term memory effects are caused by a multitude of environmental components such as temperature, trapping effects and non ideal bias networks.

Behavioural modeling is utilised to produce models of PAs that take into account the above effects and characterise them adequately while maintaining high fidelity.

The primary objective of this manuscript is to train a model combining a polynomial based model with memory and RLS error correction in order to identify an early stopping criterion for use during training of the PA model.

The Volterra model and RLS error correction were chosen due to their combined propensity for instability during training. Experimental analysis was conducted to determine and implement the early stopping criteria in this context.

The major contributions of this manuscript is presented as follows

1. A dedicated literature review for RLS stability inducing modifications.
2. The introduction of a computationally efficient method to halt RLS training before model experiences instability.

The remainder of the paper is organised as follows: Sect. 2 discusses pre-exsisting research. Sect. 3 and 4 provides an outline of Volterra modeling and conventional RLS algorithm respectively. In Sect. 5 the early stopping criteria is introduced and justified. Sect. 6 describes the methodology specific to this manuscript. Experimental measurements are presented for the validation of the approach in Sect. 7, the future works are outlined in Sect. 8 and finally the concluding remarks are outlined in Sect. 9.

## Related Work

In this section, the review of the existing works contributed towards RLS instability detection is presented with associated advantages, disadvantages, merits and limitations.

Previous papers have demonstrated alternative methods to maintain stability during training using the RLS algorithm.

Previous works by the author of [4], demonstrated that altering the RLS algorithm to include periodic regularisation and maximum and minimum eigenvalue limitations can help to maintain stability. Regularisation by padding the diagonal of the autocorelation function causes a slight degradation in dynamic range. The method of padding the autocorrelation function is relatively simple although it is not disclosed in the paper how the eigen decomposition was calculated.The disadvantages of this methodology is that extra computations are needed. Computations are needed to set the eigenvalue thresholds and to determine a suitable padding window which is sensitive to noise. Disparate accuracy was reported dependent on the eigenvalue limits and regularisation period.

Authors of [5] alter the RLS algorithm by adapting a hybrid approach of directional and exponential forgetting factorisation to implement an adaptive forgetting factor. The proposed methodology ensures stability and convergence to a minimum error. Disparate accuracy of the proposed method is reported when comparing results using alternate PAs. The results are highly dependent on *a priori* statistical PA data. The high data dependency leads to reduced accuracy of RLS estimates. Although the methodology is robust, it is not suitable for modelling strong PA non linearities. Due to the high computationally complexity of works by [5], this method suffers from latency.

In [6] two computations of DPD coefficients are performed, with one set of coefficients specifically containing peaks. The computational complexity introduced by the necessity of computing two sets of DPD coefficients is an unattractive solution. Authors of [6] report an improvement in error estimation when compared to the RLS algorithm alone but does not conclusively eliminate instability.

Research conducted by [7] propose utilising a hybrid approach adopting both RLS and Least Mean Squares (LMS) for performing DPD. The adaptive algorithm utilises RLS when the error signal is large, for quick convergence, and subsequently automate their algorithm to adopt LMS when the error is below a set threshold value. The work by [7] details an improvement of 17*dB* when compared to a system without DPD. The validation in this work is simulated. This hybrid approach for DPD produces favourable results, but a sudden increase/decrease in error in noise, as experienced in typical experimental work, could caused the error threshold to switch RLS to LMS or visa versa.

Recent research [8] presents a method to modify the RLS algorithm by applying an error threshold. The update is based on the computation of the error as seen in (8). Should the value of the error at an instantaneous time sample be above the error threshold at an particular time sample, the algorithm re-computes the error for the following time sample, omitting the previous from the calculation of the DPD coefficients. The methodology proposed by [8] reports a 30dB improvement when compared to the un-modified RLS algorithm. The validation performed in [8], using a memory depth of 2, is simulated but the methodology would be robust against spurious noise. Authors of [8] present results which indicate severe latency.

In, authors propose that computing both a variable convergence factor and variable forgetting factor improves the steady state alignment of the proposed method when compared to both the Non Linear LMS and RLS algorithm. [9] reported a 6dB mean square error improvement when compared to the RLS algorithm. Authors of [9] minimise the a priori error signal of the RLS function when determining the variable convergence factor and variable forgetting factor, which are updated by thresholding the value of the bit error rate. The authors present a look up table method to apply DPD.

Works by [4–8] and [9] all propose techniques that require additional computations and or a modification of the RLS algorithm in order to maintain the stability during training. In this work a computationally efficient approach is presented to avoid the onset of instability during model coefficient training for the RLS algorithm. The aforementioned works present alternative methods to this manuscript and predominantly focus on reducing the error - not the elimination of error. As a result of the above works altering the RLS algorithm authors, such as [7] and [8] , achieve an improved NMSE at the cost of computational complexity.

## The Volterra Model

Digital predistortion (DPD) is a technique that illicits linear behaviour of the PA by altering the magnitude and phase of an input signal. In order for DPD to create a complimentary or inverse function to eliminate non linearities introduced by a PA, behavioural modelling is used.

The Volterra series[10, 11] calculates each interaction of its inputs up to a defined order of non-linearity. As the number of inputs or the order of non-linearity increases, the number of coefficients increases rapidly and therefore increases the computational complexity [12]. Although the computational complexity is increased the Volterra Model is capable of accurately describing non linear systems with memory[13]

Behavioural modeling using the Volterra series combines numerous linear convolutions and a non linear power series, allowing the system to be modelled while incorporating memory effects[12]. Although the accuracy of the Volterra model is high the computational complexity is also high as the number of parameters to be estimated escalates rapidly as the non linear order of the model and the memory depth heightens.

A system with finite order of non-linearity with finite memory depth can be described in the time domain by eq. 1.

1 $$\begin{aligned} y(n)=\sum _{p=1}^{P}y_{p}(n) \end{aligned}$$

Where,

$$\begin{aligned} y_{p}(n)=\sum _{i_{1}=0}^{N-1} \cdots \sum _{i_{P}=0}^{N-1}h_{p}(i_{1}, \cdots ,i_{p}) \prod _{i=1}^{p}x(n-i_{r}) \end{aligned}$$

Where *x*(*n*) and *y*(*n*) is the input and output signal to the system respectively. $h_{p}(i_{1},...,i{p})$ represents the filter co-efficient expansion utilising, *p*, the highest order for the non-linearity of the Volterra series expansion. *N* represents the maximum memory tap length chosen [14].

## Theory of Conventional Recursive Least Squares (RLS) Training Algorithm

RLS is an iterative form of least mean squares that is more rapid in converging to the minimum error while training a model[15, 16]. Using RLS without limiting the input training signal length can lead to instability during training [17]. RLS has a tendency to produce unstable models. Previous literature to the best of the authors knowledge has not identified a factor that can predict the point at which the training routine will become unstable. The following mathematical analysis provides a method to predict and constrain training to prevent instability occurring.

The exponentially weighted RLS algorithm can be adequately described in terms of its cost function. Model coefficients (in this case specifically referring to the Volterra model), are adapted based on the cost function *J*(*n*), shown below in eq. 2.

2 $$\begin{aligned} J(n) = \sum _{k=1}^{n} \lambda ^{(n-k)} (d(k) - \vec {\varvec{H}}^T(n) \varvec{X}(k))^2 \end{aligned}$$

$\lambda$ is an exponentially weighted factor, $0< \lambda <1$, controlling the convergence speed of the function, referred to here, as the forgetting factor. $\lambda$ closer to 1 enables the algorithm to decay slowly, tracking signal alterations more closely. The inverse is true for $\lambda$ tending to 0. *d*(*k*), refers to the actual output signal at sample k.Filter coefficients, $\vec {\varvec{H}}(n)$, are determined such that the weighted average of the squared estimation error is minimised from time $k=1$ to $k=n$. [17]. $\varvec{X}(k)$ represents the input signal to the model at sample k.

The following equations give a mathematical description of the RLS algorithm to minimise the cost function in eq. 2 by minimising the error $\varepsilon$ to update $\vec {\varvec{H}}(n)$ and the update matrix $\varvec{C}^{-1}(n)$ in an iterative fashion , heuristically as in the conventional RLS algorithm [17] where *K*(*n*) depicts the gain vector.

3 $$\begin{aligned}&\varepsilon (n) = d(n)- \varvec{X}^{T}(n)\varvec{H}(n-1) \end{aligned}$$

4 $$\begin{aligned}&K(n) = \varvec{C}^{-1}(n) \varvec{X}(n) \end{aligned}$$

5 $$\begin{aligned}&\varvec{C}^{-1}(n) = \frac{\varvec{C}^{-1}(n-1)}{\lambda } -\frac{K(n)\varvec{X}^{T}(n)\varvec{C}^{-1}(n-1)}{\lambda } \end{aligned}$$

6 $$\begin{aligned}&\varvec{H}(n) =\varvec{H}(n-1)+K(n)\varepsilon (n) \end{aligned}$$

Where,

7 $$\begin{aligned}&\varvec{C}(n)=\sum _{k=1}^{n} \lambda ^{(n-k)} \varvec{X}(k)\varvec{X}^{\varvec{T}}(k) \end{aligned}$$

8 $$\begin{aligned}&e(n) = d(n)- \varvec{X}^{T}(n)\varvec{H}(n) \end{aligned}$$

$\varvec{C}(n)$ depicts the weighted least squares auto-correlation function, $\varvec{X}(k)\varvec{X}^{T}(k)$, of the N dimensional input vector $\varvec{X}(n)$.

When updating the RLS algorithm model coefficients as seen in eq. 6, the size of $\varvec{C}^{-1}(n)$ is determined by the total number of Volterra model coefficients given a particular memory length and model order of non linearity as defined by eq. 1. Increasing the order of non linearity and memory tap length of the Volterra model increases the size of $\varvec{C}^{-1}(n)$, thus eigen decomposition, as utilised in cited related work, can become extremely complex.

Equation 5 of the RLS training algorithm presents that the expected value of $\varvec{C}^{-1}(n)$ is a function of the auto correlation matrix. When examining an isolated sample of the signal, $\varvec{X}_{n}$, it is treated as a random variable with expected values in the form of $E\{\varvec{X} \varvec{X}^{\varvec{T}} \}$. This is used in eq. 7 and expanded in eq. 9 to illustrate the behavior of the calculation .

9 $$\begin{aligned} \varvec{R_{xx}} =\begin{bmatrix} E[\varvec{X}_{1}\varvec{X}_{1}] &{} E[\varvec{X}_{1}\varvec{X}_{2}] &{} \ldots &{}E[\varvec{X}_{1}\varvec{X}_{n}]\\ E[\varvec{X}_{2}\varvec{X}_{1}] &{} \ldots &{} &{} \vdots \\ \vdots \\ E[\varvec{X}_{n}\varvec{X}_{1}] &{} E[\varvec{X}_{n}\varvec{X}_{2}] &{} \ldots &{} E[\varvec{X}_{n}\varvec{X}_{n}]\\ \end{bmatrix} \end{aligned}$$

Equation 9 assumes that all of the components are real random vectors. Should the vectors be considered as complex values random vectors $\varvec{R_{xx}}$ must be in Hermitian form [17], which is not realisable in every Volterra model when using various memory and non linear order values.

**Fig. 1:**
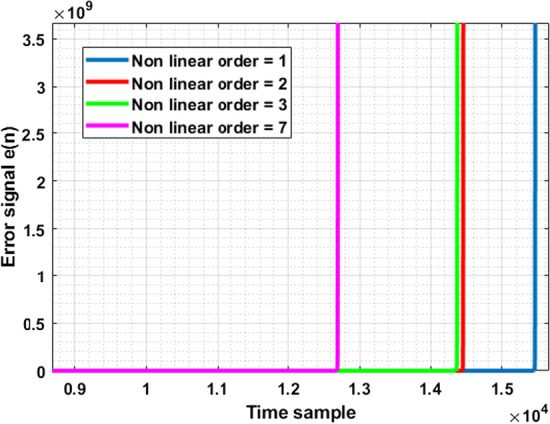
An illustration of the error signal increasing in magnitude versus time samples of the input training signal. Instability is indicated by a rapid increase in magnitude, as shown for each non linear order of the Volterra model

The model depicted in Fig. 1 was of non linear order one, two, three, and seven with a memory length of three. The model error estimate *e*(*n*), is given by $e(n) = d(n) - \hat{d}(n)$, where $\hat{d}(n)$ represents the estimated output of the model. The value of *e*(*n*) becomes extremely high abruptly at the onset of instability due to the error having reached it’s minimum as defined by RLS, resulting in a divergence from the minimum error.

Each order of non-linearity encounters the onset of instability at a different time sample for the same dataset. The calculation of minimum error is estimated utilising 9, of different sizes depending on the non linear order of the model as shown in eq. 5. Therefore there is a finite input length of training signal $\varvec{X}(n)$ that can be utilised for this model before instability occurs, regardless of the value of non-linear order, memory tap length and sampling frequency.

## Early Stopping Criterion

RLS is an iterative form of the least squares (LS) estimation. For a linear system, the LS estimate is given by

10 $$\begin{aligned} Ax = b \end{aligned}$$

The LS solution is calculates a value of *x* such that *Ax* is the closest value as possible to *b*. LS exploits the fact that $\left\| b - Ax\right\|$ is the square root of a sum of squares. Consider *A* to be an $n \times m$ matrix, *b* is in $R^m$, the LS solution of (10) is a value of *x* in $R^{n}$ such that

11 $$\begin{aligned} \left\| b - A\hat{x}\right\| \le \left\| b - Ax\right\| \end{aligned}$$

for all *x* in $R^{n}$. Considering (11) graphically in vector form the LS estimate deems that *Ax* will be in column space A (*C*(*A*)), as it is inherently limited to *C*(*A*). LS calculates a value of *x* such that *Ax* is as geometrically as close to *b* as possible in terms of distance, such that $\left\| Projection(b) - b\right\| \rightarrow 0$

12 $$\begin{aligned} A\hat{x} - b \in C_{A} \end{aligned}$$

**Fig. 2:**
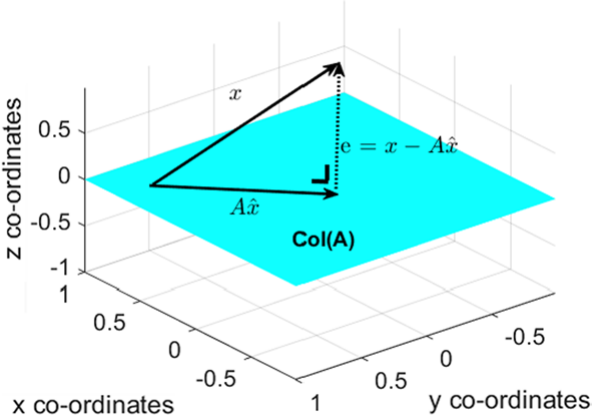
Illustration of orthogonality of LS estimation on column space A

$\hat{b}$ must be the orthogonal projection of *b* on to *Col*(*A*), for the solution of $Ax = \hat{b}$ to be valid. This entails that $Ax=\hat{b}$ is consistent and that there is a solution of $\hat{x}$ in $R^{n}$. By the orthogonal decomposition principle, the projection has the property that $b - \hat{b}$ is orthogonal to *Col*(*A*) [18].

In RLS the cost function as seen in (2) can be written as eq. 13.

13 $$\begin{aligned} J(n) = \sum _{k=1}^{n} \lambda ^{(n-k)} (d(k) - \hat{d}(n))^2 \end{aligned}$$

As illustrated above in Fig. 2$\hat{d}(n)$ must be orthogonal to *d*(*n*), a change in phase indicates a change in the $\hat{d}(n)$ projection. $\lambda ^{(n-k)}$ is inherently a scalar value. Multiplying by $\lambda ^{(n-k)}$ will not change the phase of $\hat{d}$ as the imaginary term will always be 0.

Aforementioned related works focus on improving the NMSE or adding additional computational complexity by altering the RLS algorithm. This manuscript determines a method by which to desist training of a model using RLS before instability occurs. Instability can be circumvented by a simple observation of a value that is pre-existing natively in the conventional RLS algorithm and therefore does not increase computational complexity.

## Methodology

The update matrix as in eq. 5 is calculated by the difference of two separate matrix manipulations, with one matrix manipulation containing the more current information of the autocorrelation function, consisting of the right hand side of eq. 5. Henceforth, this will be referred to as the change in update matrix and denoted it as $\triangle C^{-1} (n)$, as defined in equ. 14. Equation 5 contains an inherent flaw i.e. that a difference equation has the potential to generate eigenvalues that result in a divergence from the trajectory of minimum error [17]. To avoid the estimated output of the model diverging from the least squares error, previous authors have examined eigen analysis of the auto correlation function. This involves altering the limits specified in the auto correlation function based on the statistical analysis of the specific input training signal and, therefore, requiring individual computations for each respective training signal.

14 $$\begin{aligned} \triangle C^{-1} (n) = \frac{K(n)\varvec{X}^{T}(n) \varvec{C}^{-1}(n-1)}{\lambda } \end{aligned}$$

As RLS minimises the linear least cost function, the phase $\triangle C^{-1} (n)$ is expected to tend toward the projection of the minimum error with consistent phase, i.e the desired output and actual output are tending toward the same point in the complex plane. Significant deviations in the complex values of $\triangle C^{-1} (n)$ from the original trajectory indicates definitively the onset of instability. The phase of $\triangle C^{-1} (n)$ was chosen as the stopping criterion as it does not add any additional computational complexity.

By observing $\triangle C^{-1} (n)$ on a sample per sample basis it is possible to identify the sample point at which instability begins to occur. As the auto correlation function relates *X*(*n*) and $X(n-1)$, which contain $N-1$ common elements, and therefore should remain highly similar to previous values. Observing the first element of the matrix $\triangle C^{-1} (n)$, allows for a comprehensive measurement of the eigen vector behaviour as the diagonal values of $\triangle C^{-1} (n)$ will be identical as seen in eq. 9. In this way, a deviation in the sample to sample values in $\triangle C^{-1} (n)$ indicates a deviation from the trajectory towards the minimum error.

**Fig. 3:**
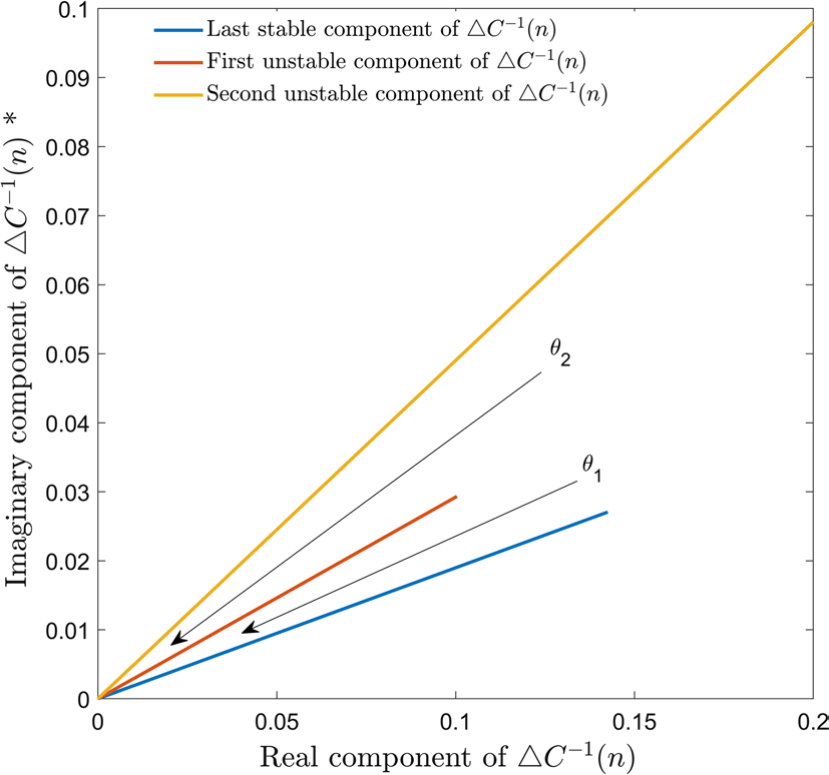
Illustration of phase discrepancy, of $\triangle C^{-1}(n)$. $\theta _{1}$ depicts the phase of the first complex value of $\triangle C^{-1} (n)$ is 0.1876 Radians. $\theta _{2}$ is 0.5278 Radians. * The limits of this figure have been truncated for aesthetic purposes. Please note the magnitude of the second unstable $\triangle C^{-1} (n)$ extends to co-ordinates $0.5891 + 0.2888i$

Figure 3 depicts how stability may be inferred from the proposed surrogate measure $\triangle C^{-1} (n)$. As previously stated, stability is indicated by the plotted vectors remaining in close proximity to the trajectory of minimum error. A change in direction and sudden increase of the magnitude of the vectors indicates that the estimate $\hat{d}(n)$, is tending away from the plane of the least squares estimation of the error.

While a simulated PA will not introduce any external errors into the model, experimental validation may incur errors such as those resulting from noise contributions. As such it is necessary to introduce a threshold into the early stopping criterion to prevent premature termination of training. The tolerance we suggest is that the phase component of $\triangle C^{-1} (n)$ should fall between $\mp 0.25$ Radians. The phase of the $\triangle C^{-1} (n)$ rises rapidly, as can be seen in Figs. 6 (a) and 3. Therefore $\mp 0.25$ Radians was considered to be a suitable prescribed tolerance as ceasing training prior to an extreme divergence of the phase component allows for the RLS algorithm preserve high fidelity of the estimated output without alteration to the RLS algorithm.

In Fig. 3 it can be seen that the model behaviour has become imbalanced i.e. the vectors have exceeded $\mp 0.25$ Radians prescribed tolerance. Once the point of convergence, or minimum error, is exceeded the eigen vectors are becoming oscillatory and increase in magnitude, i.e. attempting to point in the direction of largest variance, $\infty$ [19]. Therefore, it is beneficial to cease training once the point of convergence has been exceeded as defined by the prescribed threshold that is applied to the resulting surrogate measure obtained from the first value of the matrix given by eq. 9.

## Experimental Validation

In order to validate the early stopping criteria proposed in this work a variety of single carrier signals are sent from an AD-FMCOMMS3 evaluation board, through a Doherty PA at 2.6GHz (NXP BGA7210). The corresponding input and output signals are sampled at 30.72 MHz.

**Fig. 4:**
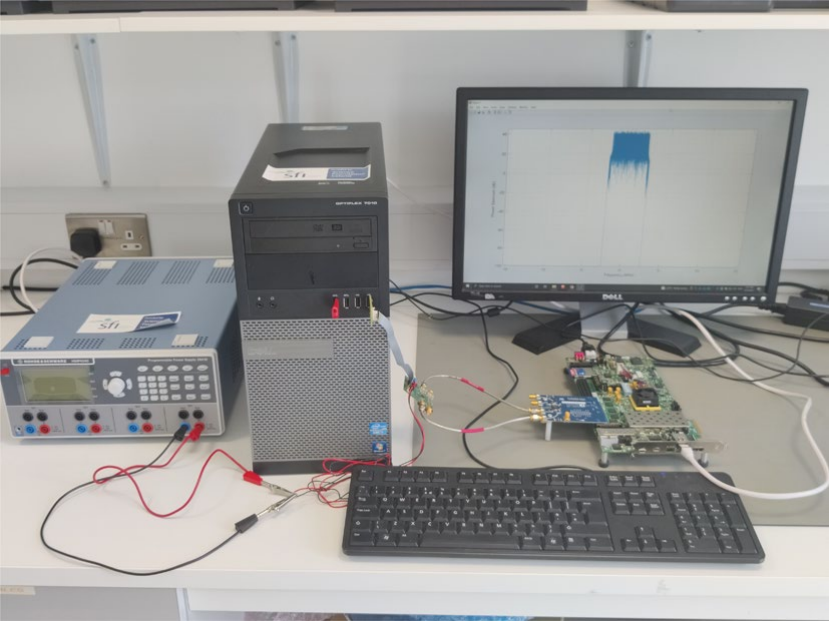
Experimental hardware setup

As can be seen in fig. 4 the hardware configuration to complete model extraction was a combination of the ZC706 and FMCOMMS3. The SMA cables were connected from TX1 to the input of the PA (NXP BGA7210) and connected back via RX1 from the output of the PA. A spectrum analyser, Rhode & Schwarz FSL, was utilised to visually inspect the various non linearities captured.

**Fig. 5:**
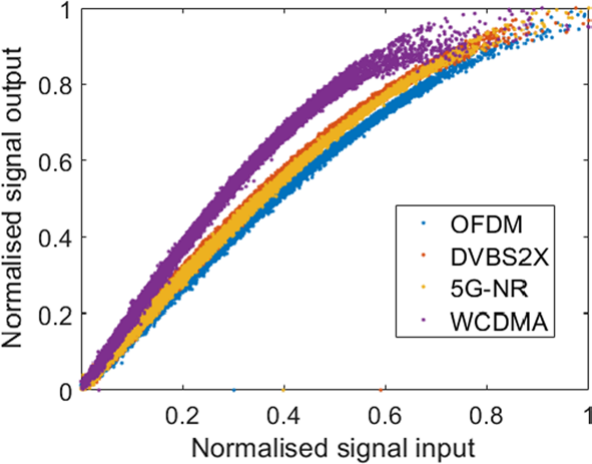
AMAM plot of examples of input output signal pairs sent and received through the PA using AD-FMCOMMS3

Various strengths of non linearity was modelled by the proposed methodology. Signal strength was increased to induce severe saturation as shown in Fig. 5. Prompting this response from the PA enabled the authors to discern the fidelity of the PA model and ensure robust modelling.

**Fig. 6:**
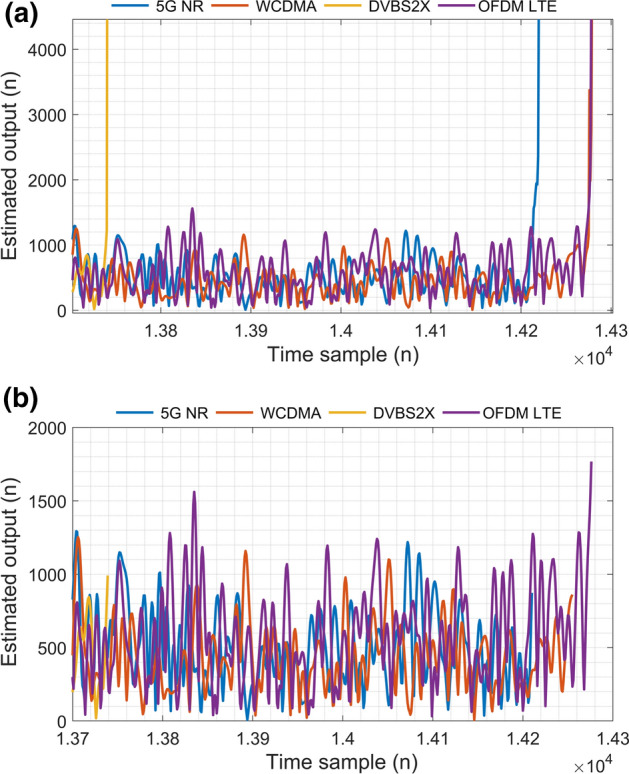
Experimentally validated output signal** a** estimated signal output without early stopping criterion and** b** with proposed early stopping criterion. It can be seen in (b) that the input training signal length has been truncated prior to the onset of instability. Let it be noted that for illustrative purposes only samples from 13700 onwards are depicted

To illustrate the onset of instability an arbitrary memory length and order of non linearity was tested. For the purposes of illustration both values were set to 2 producing Fig. 6. Each of the four modulation schemes become unstable at different time samples (n) as seen in 6 (a). DVBS2x becomes unstable at $\hbox {n}= 1.3742 \times 10^{4}$, WCDMA becomes unstable at n=$1.385 \times 10^{4}$, 5G NR becomes unstable at $\hbox {n} =1.425 \times 10^{4}$ and OFDM LTE becomes unstable at $\hbox {n} = 1.428 \times 10^{4}$.

Figure 6 (b), plots the experimentally validated output of the PA versus the estimated output of the PA model utilising the proposed algorithm. The early stopping criterion algorithm ceased training prior to instability as defined by the phase contained by the first complex value of $\triangle C^{-1} (n) > 0.25$ Radians. Both estimated outputs were compared to the experimentally validated output in terms of the Normalised Mean Square Error (NMSE) as illustrated by Table 1.

**Fig. 7:**
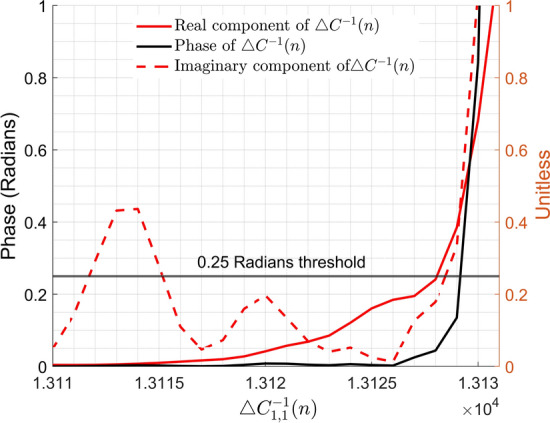
Real, Imaginary and Phase components of the change in update matrix, $\triangle C^{-1} (n)$, as the early stopping criterion is surpassed. The early stopping criterion is shown as a constant 0.25 Radians threshold that indicates instability when exceeded by the phase. Phase was chosen rather than the real or imaginary components as the indicating factor in order to maximise the input training signal length. Let it be noted the limits of this figure have been truncated for aesthetic purposes

Figure 7 illustrates the onset of instability with regard to the phase, imaginary and real component of $\triangle C^{-1} (n)$. The imaginary and phase components of the first element of $\triangle C^{-1} (n)$ diverge by a large amount close the point of onset of instability.

**Fig. 8:**
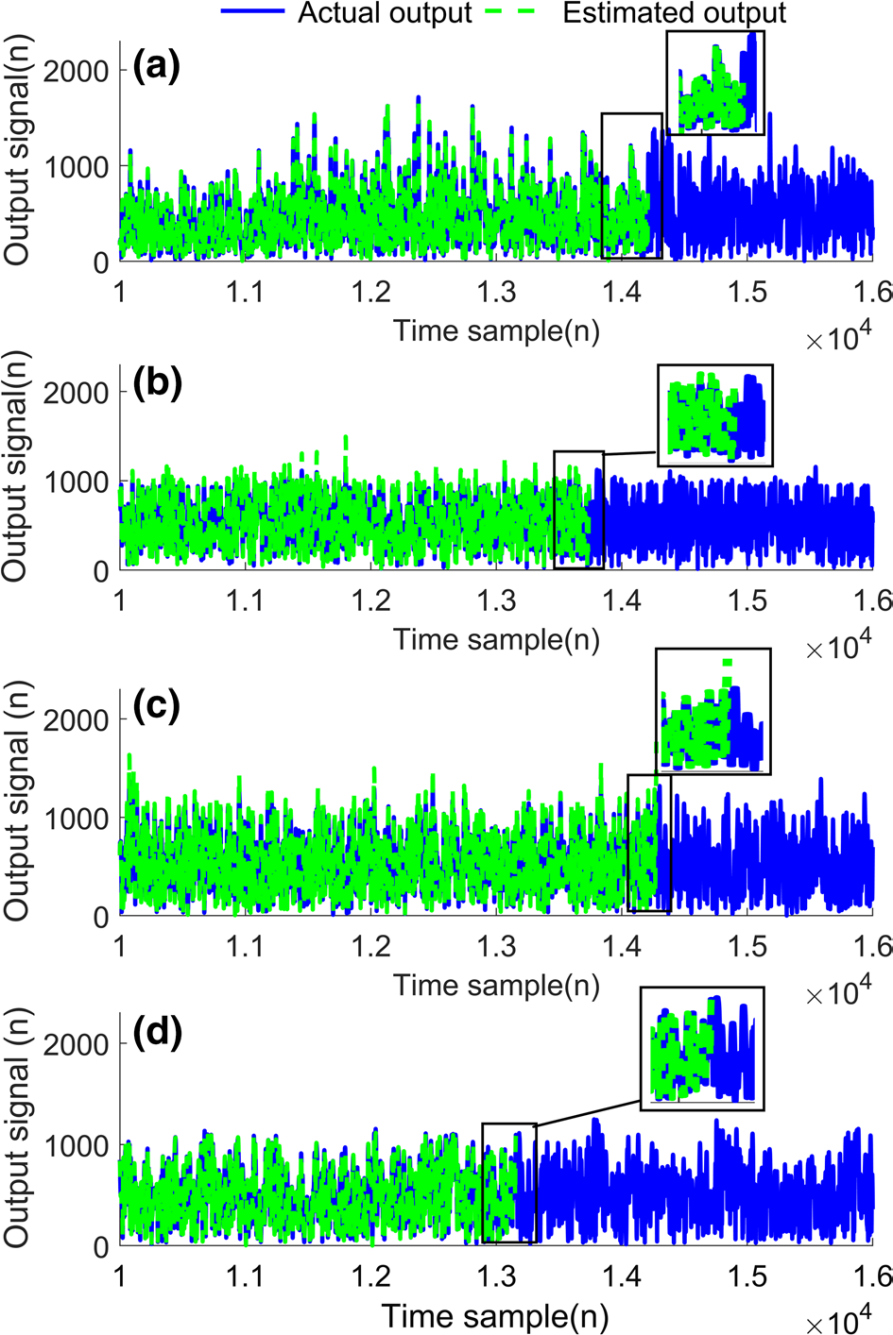
Experimental signal output versus estimated signal output of various signal standards with proposed algorithm** a** 5G-NR,** b** DVBS2X,** c** LTE OFDM and** d** WCDMA.In each of these cases it can be seen that the estimated output corresponds closely with the experimentally validated output, indicating that the applied algorithm does not negatively affect the modeling capabilities. Zoomed in sections have been provided for clarity. Let it be noted that for illustrative purposes only samples from $10^{3}$ onwards are depicted

The model utilising the proposed algorithm returned acceptable model accuracy in terms of NMSE values, as seen in Table 1. The NMSE values listed in the table show that, by utilising the early stopping criterion, the NMSE value indicates high fidelity between the estimated output and the actual output (visually illustrated in Fig. 8). Severe degradation of the NMSE values occurs rapidly after this point, as shown by the NMSE values listed for +10 and +20 samples after the early stopping criterion recommends the cessation of training. Not only will the early stopping criteria prevent the training routine producing unstable outputs, but it will also maximise the length of the input training signal. Therefore allowing the continued reduction of the model coefficient error, maximising the accuracy of the extracted model.

Autonomous control of the stopping criteria removes the possibility of experimental error through heuristic approaches. Let it be noted that this experiment was conducted with various non linear orders and memory lengths. The early stopping criteria operated as expected for all values, including memory lengths and non linear orders of disparate values. As expected from Fig. 1, the input training signal length approached instability earlier as the memory lengths and non linear orders increased.

Figure 8, plots the experimentally validated output of the model trained with distinct signal standards versus the estimated output of the model using the proposed algorithm. All signals were 5MHz bandwidth single carrier signals sent through a Doherty PA at 2.6GHz, the same experimental procedure as mentioned above.

The NMSE values shown in Table 1 for the various signal standards each show a distinct improvement where the early stopping criterion has been implemented. Three of four signals NMSE values dis-improve marginally up to ten time samples after the early stopping criterion is met. However, twenty time samples after the early stopping criterion is met, the extracted model NMSE values have dramatically degraded in all four cases. The NMSE values in Table 1 indicate that, through the application of the proposed early stopping criterion, training is terminated prior to instability occurring. This does not require alteration of the RLS algorithm rather a monitoring of a single value that is inherent to the calculation.

**Table 1:** A comparison of NMSE values when stopped using early stopping criterion(ESC), 10 samples beyond ESC, 20 samples beyond ESC

Signal Standard	ESC	ESC+10	ESC + 20
WCDMA	− 25.514 dB	− 24.737 dB	5.286 dB
DVBS2X	− 25.427 dB	− 14.459 dB	14.941 dB
LTE OFDM	− 24.904 dB	− 24.903 dB	4.118 dB
5G-NR	− 24.4079 dB	− 23.615 dB	-3.136 dB

## Further Work

The limitations with this work is associated with the evolution of future generations of telecommunications. Although RLS is utilised in this manuscript for modelling PAs, the proposed methodology could be augmented to conduct error corrections for future generations of wireless communications. Further work is planned in order to keep up to date with the progression of emerging LiFi enhancements. Optical communications systems, although transmit data predominantly via light [20], errors can occur and quickly converging error estimates could be necessary to correct or model distortions such as motion blur[21]. Another limitation related to the proposed methodology is that, unlike works by [4–8] and [9], the error of the behavioural model is halted prior to instability and inherently does not contribute to actively developing methods to improve NMSE values but instead guarantees stability.

## Conclusion

In conclusion, this paper provides an early stopping criterion to avoid the onset of instability of a polynomial model during RLS training. Experimental validation of the proposed procedure shows that the NMSE of the experimental output vs estimated output indicates high fidelity until the training instance identified by the early stopping criterion is exceeded and then it deteriorates rapidly. NMSE values are detailed in Table 1 and visually illustrated by Fig. 3, 6, and 7. Application of this early stopping procedure eliminates the need to apply supplementary computational analysis, thereby minimising the computational complexity required to guarantee a stable model while maintaining it’s accuracy. Automating this early stopping procedure is conveniently implemented, only requiring the observation of a pre-existing value that is produced by the RLS algorithm, the autocorrelation based update. This method has been experimentally validated for a high power amplifier using four signal standards namely LTE OFDM, WCDMA, DVBS2X and 5G-NR.
